# Supplementary material for: Recreating the synthesis of starch granules in yeast
Source: eLife. 2016 Nov 22;5:e15552. doi: 10.7554/eLife.15552 (PMC5119888; doi:10.7554/eLife.15552)
Supplement: Supplementary file 1. — DOI: http://dx.doi.org/10.7554/eLife.15552.022 [file elife-15552-supp1.docx]

**Supplementary File 1A.** Genotypes of yeast strains used in the present study. Wild type (WT) yeast represents *Saccharomyces cerevisiae* CEN.PK113-11C (kindly provided by Barbara A. Halkier, University of Copenhagen, Denmark). The source of all other yeast strains is this study. *X-2*, *X-4*, *XI-2*, *XII-1, XII-2 and XII-5* are gene loci from a yeast expression platform (Mikkelsen et al., 2012) chosen for stable multiple gene expression. *SS1*, *SS2*, *SS3*, *SS4*, *BE1*, *BE2*, *BE3* and *glgC*-*TM* are driven by P*_GAL1_*, ISA1 and ISA2 by the bidirectional P*_GAL10_*-P*_GAL1_*. *SS2* and *glgC*-*TM* carry C-terminal *HA* tags, *SS4* and *BE1* carry C-terminal *FLAG* tags.

| **Strain** | **Genotype** |
| --- | --- |
| **WT** | *MATa MAL2-8C SUC2 his3Δ ura3-52* |
| **A** | *MATa MAL2-8C SUC2 his3Δ XI-2::ISA1-ISA2* |
| **B** | *MATa MAL2-8C SUC2 his3Δ glc3::BE3* |
| **D** | *MATa MAL2-8C SUC2 his3Δ gdb1Δ gph1Δ glc3::BE3 gsy1::GlgC-TM gsy2::SS1* |
| **E** | *MATa MAL2-8C SUC2 his3Δ gdb1Δ gph1Δ glg1Δ glg2Δ glc3::BE3 gsy1::GlgC-TM gsy2::SS1* |
| **F** | *MATa MAL2-8C SUC2 his3Δ gdb1Δ gph1Δ glc3::BE3 gsy1::GlgC-TM gsy2::SS1 XI-2::ISA1-ISA2* |
| **G** | *MATa MAL2-8C SUC2 his3Δ gdb1Δ gph1Δ glg1Δ glg2Δ glc3::BE3 gsy1::GlgC-TM gsy2::SS1 XI-2::ISA1-ISA2* |
| **H** | *MATa MAL2-8C SUC2 his3Δ gdb1Δ gph1Δ glc3::BE3 gsy1::GlgC-TM gsy2::SS2* |
| **I** | *MATa MAL2-8C SUC2 his3Δ gdb1Δ gph1Δ glg1Δ glg2Δ glc3::BE3 gsy1::GlgC-TM gsy2::SS2* |
| **J** | *MATa MAL2-8C SUC2 his3Δ gdb1Δ gph1Δ glc3::BE3 gsy1::GlgC-TM gsy2::SS2 XI-2::ISA1-ISA2* |
| **K** | *MATa MAL2-8C SUC2 his3Δ gdb1Δ gph1Δ glg1Δ glg2Δ glc3::BE3 gsy1::GlgC-TM gsy2::SS2 XI-2::ISA1-ISA2* |
| **L** | *MATa MAL2-8C SUC2 his3Δ gdb1Δ gph1Δ glc3::BE3 gsy1::GlgC-TM gsy2::SS3* |
| **M** | *MATa MAL2-8C SUC2 his3Δ gdb1Δ gph1Δ glg1Δ glg2Δ glc3::BE3 gsy1::GlgC-TM gsy2::SS3* |
| **N** | *MATa MAL2-8C SUC2 his3Δ gdb1Δ gph1Δ glc3::BE3 gsy1::GlgC-TM gsy2::SS3 XI-2::ISA1-ISA2* |
| **O** | *MATa MAL2-8C SUC2 his3Δ gdb1Δ gph1Δ glg1Δ glg2Δ glc3::BE3 gsy1::GlgC-TM gsy2::SS3 XI-2::ISA1-ISA2* |
| **P** | *MATa MAL2-8C SUC2 his3Δ gdb1Δ gph1Δ glc3::BE3 gsy1::GlgC-TM gsy2::SS4* |
| **Q** | *MATa MAL2-8C SUC2 his3Δ gdb1Δ gph1Δ glg1Δ glg2Δ glc3::BE3 gsy1::GlgC-TM gsy2::SS4* |
| **R** | *MATa MAL2-8C SUC2 his3Δ gdb1Δ gph1Δ glc3::BE3 gsy1::GlgC-TM gsy2::SS4 XI-2::ISA1-ISA2* |
| **S** | *MATa MAL2-8C SUC2 his3Δ gdb1Δ gph1Δ glg1Δ glg2Δ glc3::BE3 gsy1::GlgC-TM gsy2::SS4 XI-2::ISA1-ISA2* |
| **T** | *MATa MAL2-8C SUC2 his3Δ gdb1Δ gph1Δ glg1Δ glg2Δ glc3::BE2 gsy1::GlgC-TM gsy2::SS4 XI-2::ISA1-ISA2* |
| **U** | *MATa MAL2-8C SUC2 his3Δ glc3Δ gsy1::GlgC-TM gsy2::SS3* |
| **V** | *MATa MAL2-8C SUC2 his3Δ glc3::BE1 gsy1::GlgC-TM gsy2::SS3* |
| **W** | *MATa MAL2-8C SUC2 his3Δ glc3::BE1 gsy1::GlgC-TM gsy2::SS3* |
| **X** | *MATa MAL2-8C SUC2 his3Δ glc3::BE2 gsy1::GlgC-TM gsy2::SS3* |
| **Y** | *MATa MAL2-8C SUC2 his3Δ glc3::BE3 gsy1::GlgC-TM gsy2::SS3* |
| **0** | *MATa MAL2-8C SUC2 his3Δ gdb1Δ gph1Δ glg1Δ glg2Δ glc3::BE3 XI-2::BE2 gsy1::GlgC-TM gsy2::SS1* |
| **1** | *MATa MAL2-8C SUC2 his3Δ gdb1Δ gph1Δ glg1Δ glg2Δ glc3::BE3 XII-2::BE2 gsy1::GlgC-TM gsy2::SS1 XI-2::ISA1-ISA2* |
| **2** | *MATa MAL2-8C SUC2 his3Δ gdb1Δ gph1Δ glg1Δ glg2Δ glc3::BE3 XII-2::BE2 gsy1::GlgC-TM gsy2::SS2* |
| **3** | *MATa MAL2-8C SUC2 his3Δ gdb1Δ gph1Δ glg1Δ glg2Δ glc3::BE3 XII-2::BE2 gsy1::GlgC-TM gsy2::SS2 XI-2::ISA1-ISA2* |
| **4** | *MATa MAL2-8C SUC2 his3Δ gdb1Δ gph1Δ glg1Δ glg2Δ glc3::BE3 XI-2::BE2 gsy1::GlgC-TM gsy2::SS3* |
| **5** | *MATa MAL2-8C SUC2 his3Δ gdb1Δ gph1Δ glg1Δ glg2Δ glc3::BE3 XII-2::BE2 gsy1::GlgC-TM gsy2::SS3 XI-2::ISA1-ISA2* |
| **6** | *MATa MAL2-8C SUC2 his3Δ gdb1Δ gph1Δ glg1Δ glg2Δ glc3::BE3 XI-2::BE2 gsy1::GlgC-TM gsy2::SS4* |
| **7** | *MATa MAL2-8C SUC2 his3Δ gdb1Δ gph1Δ glg1Δ glg2Δ glc3::BE3 XII-2::BE2 gsy1::GlgC-TM gsy2::SS4 XI-2::ISA1-ISA2* |
| **8** | *MATa MAL2-8C SUC2 his3Δ gdb1Δ gph1Δ glg1Δ glg2Δ glc3::BE3 XII-2::BE2 gsy1::GlgC-TM gsy2::SS1 XI-2::SS2* |
| **9** | *MATa MAL2-8C SUC2 his3Δ gdb1Δ gph1Δ glg1Δ glg2Δ glc3::BE3 XII-2::BE2 gsy1::GlgC-TM gsy2::SS1 X-4::SS2*  *XI-2::ISA1-ISA2* |
| **10** | *MATa MAL2-8C SUC2 his3Δ gdb1Δ gph1Δ glg1Δ glg2Δ glc3::BE3 XI-2::BE2 gsy1::GlgC-TM X-2::SS1 gsy2::SS3* |
| **11** | *MATa MAL2-8C SUC2 his3Δ gdb1Δ gph1Δ glg1Δ glg2Δ glc3::BE3 XII-2::BE2 gsy1::GlgC-TM X-2::SS1 gsy2::SS3*  *XI-2::ISA1-ISA2* |
| **12** | *MATa MAL2-8C SUC2 his3Δ gdb1Δ gph1Δ glg1Δ glg2Δ glc3::BE3 XI-2::BE2 gsy1::GlgC-TM gsy2::SS1 XII-1::SS4* |
| **13** | *MATa MAL2-8C SUC2 his3Δ gdb1Δ gph1Δ glg1Δ glg2Δ glc3::BE3 XII-2::BE2 gsy1::GlgC-TM gsy2::SS1 XII-1::SS4*  *XI-2::ISA1-ISA2* |
| **14** | *MATa MAL2-8C SUC2 his3Δ gdb1Δ gph1Δ glg1Δ glg2Δ glc3::BE3 XII-2::BE2 gsy1::GlgC-TM XI-2::SS2 gsy2::SS3* |
| **15** | *MATa MAL2-8C SUC2 his3Δ gdb1Δ gph1Δ glg1Δ glg2Δ glc3::BE3 XII-2::BE2 gsy1::GlgC-TM X-4::SS2 gsy2::SS3*  *XI-2::ISA1-ISA2* |
| **16** | *MATa MAL2-8C SUC2 his3Δ gdb1Δ gph1Δ glg1Δ glg2Δ glc3::BE3 XII-2::BE2 gsy1::GlgC-TM XI-2::SS2 gsy2::SS4* |
| **17** | *MATa MAL2-8C SUC2 his3Δ gdb1Δ gph1Δ glg1Δ glg2Δ glc3::BE3 XII-2::BE2 gsy1::GlgC-TM X-4::SS2 gsy2::SS4*  *XI-2::ISA1-ISA2* |
| **18** | *MATa MAL2-8C SUC2 his3Δ gdb1Δ gph1Δ glg1Δ glg2Δ glc3::BE3 XI-2::BE2 gsy1::GlgC-TM gsy2::SS3 XII-1::SS4* |
| **19** | *MATa MAL2-8C SUC2 his3Δ gdb1Δ gph1Δ glg1Δ glg2Δ glc3::BE3 XII-2::BE2 gsy1::GlgC-TM gsy2::SS3 XII-1::SS4*  *XI-2::ISA1-ISA2* |
| **20** | *MATa MAL2-8C SUC2 his3Δ gdb1Δ gph1Δ glg1Δ glg2Δ glc3::BE3 XII-2::BE2 gsy1::GlgC-TM X-2::SS1 XI-2::SS2 gsy2::SS3* |
| **21** | *MATa MAL2-8C SUC2 his3Δ gdb1Δ gph1Δ glg1Δ glg2Δ glc3::BE3 XII-2::BE2 gsy1::GlgC-TM X-2::SS1 X-4::SS2 gsy2::SS3 XI-2::ISA1-ISA2* |
| **22** | *MATa MAL2-8C SUC2 his3Δ gdb1Δ gph1Δ glg1Δ glg2Δ glc3::BE3 XII-2::BE2 gsy1::GlgC-TM gsy2::SS1 XI-2::SS2 XII-1::SS4* |
| **23** | *MATa MAL2-8C SUC2 his3Δ gdb1Δ gph1Δ glg1Δ glg2Δ glc3::BE3 XII-2::BE2 gsy1::GlgC-TM gsy2::SS1 X-4::SS2 XII-1::SS4 XI-2::ISA1-ISA2* |
| **24** | *MATa MAL2-8C SUC2 his3Δ gdb1Δ gph1Δ glg1Δ glg2Δ glc3::BE3 XI-2::BE2 gsy1::GlgC-TM X-2::SS1 gsy2::SS3 XII-1::SS4* |
| **25** | *MATa MAL2-8C SUC2 his3Δ gdb1Δ gph1Δ glg1Δ glg2Δ glc3::BE3 XII-2::BE2 gsy1::GlgC-TM X-2::SS1 gsy2::SS3 XII-1::SS4 XI-2::ISA1-ISA2* |
| **26** | *MATa MAL2-8C SUC2 his3Δ gdb1Δ gph1Δ glg1Δ glg2Δ glc3::BE3 XII-2::BE2 gsy1::GlgC-TM XI-2::SS2 gsy2::SS3 XII-1::SS4* |
| **27** | *MATa MAL2-8C SUC2 his3Δ gdb1Δ gph1Δ glg1Δ glg2Δ glc3::BE3 XII-2::BE2 gsy1::GlgC-TM X-4::SS2 gsy2::SS3 XII-1::SS4 XI-2::ISA1-ISA2* |
| **28** | *MATa MAL2-8C SUC2 his3Δ gdb1Δ gph1Δ glg1Δ glg2Δ glc3::BE3 XII-2::BE2 gsy1::GlgC-TM X-2::SS1 XI-2::SS2 gsy2::SS3 XII-1::SS4* |
| **29** | *MATa MAL2-8C SUC2 his3Δ gdb1Δ gph1Δ glg1Δ glg2Δ glc3::BE3 XII-2::BE2 gsy1::GlgC-TM X-2::SS1 X-4::SS2 gsy2::SS3 XII-1::SS4 XI-2::ISA1-ISA2* |
| **30** | *MATa MAL2-8C SUC2 his3Δ gdb1Δ gph1Δ glg1Δ glg2Δ glc3::BE3 XII-2::BE2 gsy1::GlgC-TM X-2::SS1 X-4::SS2 gsy2::SS3 XII-1::SS4 XI-2::ISA1-ISA2 XII-5::BE1* |

**Supplementary File 1B.** Plasmids used in the present study. Vectors from the yeast expression platform (Mikkelsen et al., 2012) (pX-2, pX-3, pX-4, pXI-2, pXII-1, pXII-2 and pXII-5) were kindly provided by Barbara A. Halkier, University of Copenhagen, Denmark. CDS, coding sequence; AA, amino acids; cTP, chloroplast transit peptide; ATG, start codon.

| **Vector Name** | **Description** | **Source** |
| --- | --- | --- |
| pX-2 | Yeast integration vector with *ADH1* and *CYC1* terminators targeting locus 2 on chromosome X (yeast expression platform) | B. Halkier |
| pX-3 | As pX-2, but targeting locus 3 on chromosome X | B. Halkier |
| pX-4 | As pX-2, but targeting locus 4 on chromosome X | B. Halkier |
| pXI-2 | As pX-2, but targeting locus 2 on chromosome XI | B. Halkier |
| pXII-1 | As pX-2, but targeting locus 1 on chromosome XII | B. Halkier |
| pXII-2 | As pX-2, but targeting locus 2 on chromosome XII | B. Halkier |
| pXII-5 | As pX-2, but targeting locus 5 on chromosome XII | B. Halkier |
| pGSY1 | As pX-2, but targeting *GSY1* locus (rendering *GSY1* dysfunctional) | This study |
| pGSY2 | As pGSY1, but targeting *GSY2* locus (rendering *GSY2* dysfunctional) | This study |
| pGLC3 | As pGSY1, but targeting *GLC3* locus (rendering *GLC3* dysfunctional) | This study |
| pGLG1 | As pGSY1, but targeting *GLG1* locus (rendering *GLG1* dysfunctional) | This study |
| pGLG2 | As pGSY1, but targeting *GLG2* locus (rendering *GLG2* dysfunctional) | This study |
| pGPH1 | As pGSY1, but targeting *GPH1* locus (rendering *GPH1* dysfunctional) | This study |
| pGDB1 | As pGSY1, but targeting *GDB1* locus (rendering *GDB1* dysfunctional) | This study |
| pGSY1_P*_GAL1_*-*glgC*-*TM*-*HA* | P*_GAL1_* fused to CDS of *glgC-TM* with C-terminal *HA* tag in pGSY1 | This study |
| pGSY2_ P*_GAL1_*-*SS1* | P*_GAL1_* fused to CDS of *SS1* (w/o 49 AA cTP; + ATG) in pGSY2 | This study |
| pX-2_ P*_GAL1_*-*SS1* | P*_GAL1_* fused to CDS of *SS1* (w/o 49 AA cTP; + ATG) in pX-2 | This study |
| pGSY2_ P*_GAL1_*-*SS2*-*HA* | P*_GAL1_* fused to CDS of *SS2* (codon optimized; w/o 55 AA cTP; + ATG and Ala) with C-terminal *HA* tag in pGSY2 | This study |
| pX-4_ P*_GAL1_*-*SS2*-*HA* | P*_GAL1_* fused to CDS of *SS2* (codon optimized; w/o 55 AA cTP; + ATG and Ala) with C-terminal *HA* tag in pX-4 | This study |
| pXI-2_P*_GAL1_*-*SS2*-*HA* | P*_GAL1_* fused to CDS of *SS2* (codon optimized; w/o 55 AA cTP; + ATG and Ala) with C-terminal *HA* tag in pXI-2 | This study |
| pGSY2_P*_GAL1_*-*SS3* | P*_GAL1_* fused to CDS of *SS3* (w/o 37 AA cTP; + ATG) in pGSY2 | This study |
| pGSY2_ P*_GAL1_*-*SS4-FLAG* | P*_GAL1_* fused to CDS of *SS4* (w/o 36 AA cTP; + ATG and Gly) with C-terminal *FLAG* tag in pGSY2 | This study |
| pXII-1_ P*_GAL1_*-*SS4-FLAG* | P*_GAL1_* fused to CDS of *SS4* (w/o 36 AA cTP; + ATG and Gly) with C-terminal *FLAG* tag in pXII-1 | This study |
| pGLC3_P*_GAL1_*-*BE1* | P*_GAL1_* fused to CDS of *BE1* (w/o 49 AA cTP; + ATG) with C-terminal *FLAG* tag in pGLC3 | This study |
| pXII-5_P*_GAL1_*-*BE1* | P*_GAL1_* fused to CDS of *BE1* (w/o 49 AA cTP; + ATG) with C-terminal *FLAG* tag in pGLC3 | This study |
| pGLC3_P*_GAL1_*-*BE2* | P*_GAL1_* fused to CDS of *BE2* (w/o 48 AA cTP; + ATG + Ala) in pGLC3 | This study |
| pXII-2_P*_GAL1_*-*BE2* | P*_GAL1_* fused to CDS of *BE2* (w/o 48 AA cTP; + ATG + Ala) in pXII-2 | This study |
| pXI-2_P*_GAL1_*-*BE2* | P*_GAL1_* fused to CDS of *BE2* (w/o 48 AA cTP; + ATG + Ala) in pXI-2 | This study |
| pGLC3_P*_GAL1_*-*BE3* | P*_GAL1_* fused to CDS of *BE3* (w/o 38 AA cTP; + ATG + Ala) in pGLC3 | This study |
| pXI-2_*ISA1*-P*_GAL10_*- P*_GAL1_*-*ISA2* | P*_GAL10_* fused to CDS of *ISA1* (w/o 43 AA cTP: + ATG) and P*_GAL1_* fused to CDS of *ISA2* (w/o 27 AA cTP: + ATG) in pXI-2 | This study |

**Supplementary File 1C.** Primers used for cloning of constructs. Recombination sites “up” and “down” refer to sequences on the yeast integration vectors flanking the expression cassettes up- or downstream, respectively. These sites target the DNA to specific (homologous) regions on the yeast chromosome. Restriction sites for restriction-digest based cloning are shown in red, fusion stretches for USER cloning in green. ATG start codons of coding sequences are underlined and stop codons are depicted in bold. gDNA, genomic DNA; WT, CEN-PK113-11C wild-type *S. cerevisiae*; P, plasmid; CDS, coding sequence; AA, amino acids; cTP, chloroplast transit peptide; CO, codon-optimized for *S. cerevisiae*.

| **Forward Primer Name** | **Sequence (5' - 3')** | **Reverse Primer Name** | **Sequence (5' - 3')** | **Template** | **Purpose** |
| --- | --- | --- | --- | --- | --- |
| GSY1 up fw | CCTAGGCAGACGTATCGTTGTCATCATCGTC | GSY1 up rv | GCATTCCATGCCCGCGGCTGTCACTTGGGTGTTTTTC | gDNA (WT) | Cloning of *GSY1* recombination site "up" |
| GSY1 down fw | CCTGCAGGACTTCAAGACAGAGTAAATACCACC | GSY1 down rv | TGATCAGGCGCGCCGTAAACGGAATCTTTCAGGGGCAC | gDNA (WT) | Cloning of *GSY1* recombination site "down" |
| GSY2 up fw | CCTAGGGATATCTGCACATGG | GSY2 up rv | CCGCGGAGTAAATACCACCAACC | gDNA (WT) | Cloning of *GSY2* recombination site "up" |
| GSY2 down fw | CCTGCAGGCTGAGGTGAGGGCGTTAG | GSY2 down rv | GGCGCGCCTTGAAACGACGATCCAC | gDNA (WT) | Cloning of *GSY2* recombination site "down" |
| GLC3 up fw | CCTAGGCTTTCACTATCTCTTCCGCAATACC | GLC3 up rv | CCGCGGTTTCAACCATGGATCAAATTC | gDNA (WT) | Cloning of *GLC3* recombination site "up" |
| GLC3 down fw | CCTGCAGGTGGTACTGGTTTCGACTACAG | GLC3 down rv | GGCGCGCCTGTTTCGTGTTCAGCCACTTATG | gDNA (WT) | Cloning of *GLC3* recombination site "down" |
| GLG1 up fw | TTCCTAGGATTTTCGATGAAGCGTTTTAGG | GLG1 up rv | AACCGCGGAATAGAGCAATGTGGCAATAG | gDNA (WT) | Cloning of *GLG1* recombination site "up" |
| GLG1 down fw | CCTGCAGGAGAAACGAAGACGAGTGC | GLG1 down rv | GGCGCGCCTACAAATGTCAGGGCTGCG | gDNA (WT) | Cloning of *GLG1* recombination site "down" |
| GLG2 up fw | AACCTAGGGGAAGTCGTTTATTCGACG | GLG2 up rv | AACCGCGGTAAATAGTCCCGTGAATACAG | gDNA (WT) | Cloning of *GLG2* recombination site "up" |
| GLG2 down fw | CCTGCAGGCACGTTGACCTGGATATAACC | GLG2 down rv | GGCGCGCCACGTTGTTGAATGGCAG | gDNA (WT) | Cloning of *GLG2* recombination site "down" |
| GPH1 up fw | AACCTAGGAAGCTCTGCGAGATGCAGCCAG | GPH1 up rv | TTCCGCGGAGAAGTAGGTTCTTCGGTTATC | gDNA (WT) | Cloning of *GPH1* recombination site "up" |
| GPH1 down fw | CCTGCAGGACTGCTGGTACTGAAGCGTCTG | GPH1 down rv | GGCGCGCCACGTTTTGTCACTGTCTCGC | gDNA (WT) | Cloning of *GPH1* recombination site "down" |
| GDB1 up fw | TGCCCTAGGGTAAAAACATTAGG | GDB1 up rv | CCGCGGTATCCGACAAACGTAGCAG | gDNA (WT) | Cloning of *GDB1* recombination site "up" |
| GDB1 down fw | CCTGCAGGCAATGCAAGCCTGGTCC | GDB1 down rv | GGCGCGCCCTGGTGATGCACATGC | gDNA (WT) | Cloning of *GDB1* recombination site "down" |
| USER pGAL1 fw | CGTGCGAUGTACGGATTAGAAGCCGCC | USER pGAL1 rv | ATAGTATTACGGAUCCGGGGTTTTTTCTCC | P containing P*_GAL10_*-P*_GAL1_* | USER cloning of P*_GAL1_* (for *glgC-TM-HA*, *BE2*, *BE3*, *SS1*, *SS3*, *SS4*-*FLAG*) |
| USER pGAL10 fw | ATTGTAGATUAGTGAGGGTTGAATTCG | USER pGAL1 rv | ATAGTATTACGGAUCCGGGGTTTTTTCTCC | P containing P*_GAL10_*-P*_GAL1_* | USER cloning of P*_GAL10_*-P*_GAL1_* (for *ISA1/ISA2*) |
| USER pGAL1 fw | CGTGCGAUGTACGGATTAGAAGCCGCC | USER pGAL1_2 rv | ATTATAGTTTTTUCTCCTTGACG | gDNA (WT) | USER cloning of P*_GAL1_* as in CEN.PK113-11C (for *SS2-HA* and *BE1-FLAG*) |
| USER GlgC-TM fw | ATCCGTAATACTAUGTCTGTTAGTTTAGAGAAGAAC | USER HA rv | CACGCGAU**TTA**AGCGTAATCTGGAACATCG | P containing CDS of *glgC-TM-HA* | USER cloning of *glgC-TM-HA* (full length with C-terminal *HA* tag) |
| USER SS1 fw | ATCCGTAATACTAUGTCTTCTTCCTTCTCCGGTGAC | USER SS1 rv | CACGCGAU**CTA**GCTGACATAGGGAGGGTC | P containing CDS of *SS1* | USER cloning of *SS1* (w/o 49 AA cTP; + ATG) |
| USER AtSS2_CO fw | AAAAAACTATAAUGGCTTGCGTTTCTAGAGTTGAGGCTTC | USER HA rv | CACGCGAU**TTA**AGCGTAATCTGGAACATCG | P containing CDS of CO *SS2-HA* | USER cloning of codon-optimized *SS2-HA* (w/o 55 AA cTP; + ATG, Ala; C-terminal *HA* tag) |
| USER SS3 fw | ATCCGTAATACTAUGGGAAGTGCTCAGAAAAG | USER SS3 rv | CACGCGAU**TTA**CTTGCGTGCAGAGTGATAG | P containing CDS of *SS3* | USER cloning of *SS3* (w/o 37 AA cTP; + ATG) |
| USER SS4 fw | ATCCGTAATACTAUGGGCCGATTAGTTTCTACTTCGTG | USER FLAG rv | CACGCGAU**CTA**CTTGTCATCGTCATCTTTATAATC | P containing CDS of *SS4-FLAG* | USER cloning of *SS4-FLAG* (w/o 36 AA cTP; + ATG, Gly; C-terminal *FLAG* tag) |
| USER BE1 fw | AAAAAACTATAAUGCAG GAGAAACAGAAAAAGAAGAGTC | USER FLAG rv | CACGCGAU**CTA**CTTGTCATCGTCATCTTTATAATC | P containing CDS of BE1*-FLAG* | USER cloning of BE1*-FLAG* (w/o 49 AA cTP; + ATG, C-terminal *FLAG* tag) |
| USER BE2 fw | ATCCGTAATACTAUGGCTCAATCTGCGGAGTTTGATTC | USER BE2 rv | CACGCGAU**CTA**ATCGTGGTTTGCTAAAGC | P containing CDS of *BE2* | USER cloning of *BE2* (w/o 48 AA cTP; + ATG + Ala) |
| USER BE3 fw | ATCCGTAATACTAUGGCTTCTCTGAGGAAGGACTCTCG | USER BE3 rv | CACGCGAU**CTA**AACATCTTCGGGTAACAGG | P containing CDS of *BE3* | USER cloning of *BE3* (w/o 38 AA cTP; + ATG + Ala) |
| USER ISA1(-) fw | CGTGCGAU**TCA**GGGGTCTTTAATTGGTG | USER ISA1 (-) rv | AATCTACAAUGGCAAAGGACAGAAGAAGCAACG | P containing CDS of *ISA1* | USER cloning of *ISA1* (w/o 43 AA cTP: + ATG) |
| USER ISA2 fw | ATCCGTAATACTAUGGCAAGGCTTTTTACTGGTAGG | USER ISA2 rv | CACGCGAU**CTA**AGCGGTAGTATTGATGG | P containing CDS of *ISA2* | USER cloning of *ISA2* (w/o 27 AA cTP: + ATG) |

**Supplementary File 1D.** Primer combinations for genotyping of yeast strains. Expected product lengths (in base pairs) are given for lines without modification at the respective locus (WT), transgenic (TG) lines with intact *URA3* marker and transgenic lines after excision of the *URA3* marker [TG (*ura3Δ*)] at the respective locus, respectively. The transforming plasmids (constructs) and the associated loci are described in **Supplementary File 1B**. The presence or absence of all expression constructs irrespective of location was additionally confirmed in final lines using a promoter-specific and a gene-specific primer (last eight rows). Primers named "up out fw" align upstream of the recombination site "up" (i.e. on genomic DNA of both WT and transgenic lines), primers named "down out rv" align downstream of the recombination site "down" (again on genomic DNA of both WT and transgenics). In the case of *GLG1* and *GLG2*, these primers anneal to a region far downstream of the insertion sites due to high homology in the downstream genes of *GLG1* and *GLG2* in order to ensure specificity. The forward primer "DR fw" binds to the *CYC1* terminator sequence on the yeast integration vector, i.e. upstream of the *URA3* gene, thus allowing to confirm the excision of that gene by product length (**Figure 1-figure supplement 1C**). The forward primer "yeast fw2" anneals between the *URA3* and recombination site "down" on the integration vector. The reverse primer “yeast rv2” anneals to the *CYC1* terminator sequence on the yeast integration vector. All other primers are specific for the coding sequences of g*lgC*-*TM,* Arabidopsis genes or P*_GAL_* promoters, respectively, as indicated in brackets.

|  |  |  |  |  |  | | | |  |
| --- | --- | --- | --- | --- | --- | --- | --- | --- | --- |
| **Construct** | **Forward Primer Name** | **Sequence (5' - 3')** | **Reverse Primer Name** | **Sequence (5' - 3')** | **WT** | **TG (*URA3*)** | **TG (*ura3****Δ***)** |  |  |
| **pGLG1** | GLG1 up out fw | TGATTCTTTGAACCGTTTGGCTC | yeast rv2 | ATGTTACATGCGTACACG | - | 1027 | 1027 |  |  |
|  | DR fw | TGTACAGACGCGTGTACGCATG | GLG1 far down out rv | CGTGCCGAGAGTCTTTTGGAC | - | 3927 | 2534 |  |  |
|  | GLG1 up out fw | TGATTCTTTGAACCGTTTGGCTC | GLG1 far down out rv | CGTGCCGAGAGTCTTTTGGAC | 4150 | 4926 | 3533 |  |  |
|  | yeast fw2 | GTACCCAATTCGCCCTATAG | GLG1 far down out rv | CGTGCCGAGAGTCTTTTGGAC | - | 2267 | 2267 |  |  |
| **pGLG2** | GLG2 up out fw | GCGCCATTCCAGGTGAATACATC | yeast rv2 | ATGTTACATGCGTACACG | - | 1001 | 1001 |  |  |
|  | yeast fw2 | GTACCCAATTCGCCCTATAG | GLG2 far down out rv | CCAAAGCGACCAGTAAAACAGC | - | 2086 | 2086 |  |  |
|  | GLG2 up out fw | GCGCCATTCCAGGTGAATACATC | GLG2 far down out rv | CCAAAGCGACCAGTAAAACAGC | 3515 | 4720 | 3327 |  |  |
|  | DR fw | TGTACAGACGCGTGTACGCATG | GLG2 far down out rv | CCAAAGCGACCAGTAAAACAGC | - | 3747 | 2354 |  |  |
| **pGPH1** | GPH1 up out fw | AATGAAATATACCCCGGTGCTTG | yeast rv2 | ATGTTACATGCGTACACG | - | 1060 | 1060 |  |  |
|  | GPH1 up out fw | AATGAAATATACCCCGGTGCTTG | GPH1 down out rv | GGATACAACACAGCGGTTATAGAC | 3810 | 3772 | 2379 |  |  |
|  | DR fw | TGTACAGACGCGTGTACGCATG | GPH1 down out rv | GGATACAACACAGCGGTTATAGAC | - | 2740 | 1347 |  |  |
| **pGDB1** | GDB1 up out fw | CTGGTCCATTCTGTTCTTCTACG | yeast rv2 | ATGTTACATGCGTACACG | - | 969 | 969 |  |  |
|  | DR fw | TGTACAGACGCGTGTACGCATG | GDB1 down out rv | CGAAAACCTGAGAGAACAGGTGG | - | 2326 | 933 |  |  |
|  | GDB1 up out fw | CTGGTCCATTCTGTTCTTCTACG | GDB1 down out rv | CGAAAACCTGAGAGAACAGGTGG | 2982 | 3365 | 1972 |  |  |
| **pGLC3** | GLC3 up out fw | CATACTATACCACACGTACGAC | yeast rv2 | ATGTTACATGCGTACACG | - | 956 | 956 |  |  |
|  | DR fw | TGTACAGACGCGTGTACGCATG | GLC3 down out rv | AGTACCAGCTTTTTCGACACC | - | 2413 | 1020 |  |  |
|  |  |  |  |  |  |  |  |  |  |
| **pGSY1_ P*_GAL1_*-*GlgC*-*TM*-*HA*** | GSY1 up out fw | GCAAAGTATCCCACGTAAAAGGTTCC | GlgCTM rv (in *GlgC*-*TM*-*HA*) | AATGACCAGCCGCGCTGAATGTGC | - | 1778 | 1778 |  |  |
|  | GSY1 up out fw | GCAAAGTATCCCACGTAAAAGGTTCC | GSY1 down out rv | GAAAGCTTAAGCGCGCTATGTAAC | 2963 | 5510 | 4117 |  |  |
|  | DR fw | TGTACAGACGCGTGTACGCATG | GSY1 down out rv | GAAAGCTTAAGCGCGCTATGTAAC | - | 2546 | 1153 |  |  |
| **pGSY2_ P*_GAL1_*-*SS1*** | GSY2 up out fw | GTGTGTTGAGCAAAGCACGCCATCAC | 878 (in *SS1*) | TACGCCAAAGTCAGCCATTACAA | - | 1646 | 1646 |  |  |
|  | GSY2 up out fw | GTGTGTTGAGCAAAGCACGCCATCAC | GSY2 down out rv | GAGACCCATTCTTTTCCAGTCCAG | 2467 | 5950 | 4557 |  |  |
|  | DR fw | TGTACAGACGCGTGTACGCATG | GSY2 down out rv | GAGACCCATTCTTTTCCAGTCCAG | - | 2355 | 962 |  |  |
|  | 948 (in SS1) | GCAATGAGATACGGAACCATTC | GSY2 down out rv | GAGACCCATTCTTTTCCAGTCCAG | - | 2891 | 1498 |  |  |
| **pX-2_ P*_GAL1_*-*SS1*** | X-2 up out fw | ATGATGACTGTCCGCTGGAGC | 878 (in *SS1*) | TACGCCAAAGTCAGCCATTACAA | - | 1621 | 1621 |  |  |
|  | X-2 up out fw | ATGATGACTGTCCGCTGGAGC | X-2 down out rv | GTGAGCCTCTTACCTGTTTGG | 1434 | 5982 | 4589 |  |  |
|  | DR fw | TGTACAGACGCGTGTACGCATG | X-2 down out rv | GTGAGCCTCTTACCTGTTTGG | - | 2390 | 997 |  |  |
|  | 948 (in SS1) | GCAATGAGATACGGAACCATTC | X-2 down out rv | GTGAGCCTCTTACCTGTTTGG | - | 2948 | 1555 |  |  |
| **pGSY2_ P*_GAL1_*-*SS2*-*HA*** | GSY2 up out fw | GTGTGTTGAGCAAAGCACGCCATCAC | SS2 CO rv2 (in *SS2*-*HA*) | GGTTTGTAAGGTGGGTCAACAG | - | 1920 | 1920 |  |  |
|  | GSY2 up out fw | GTGTGTTGAGCAAAGCACGCCATCAC | GSY2 down out rv | GAGACCCATTCTTTTCCAGTCCAG | 2467 | 6255 | 4862 |  |  |
|  | DR fw | TGTACAGACGCGTGTACGCATG | GSY2 down out rv | GAGACCCATTCTTTTCCAGTCCAG | - | 2453 | 1060 |  |  |
| **pX-4_ P*_GAL1_*-*SS2*-*HA*** | X-4 up out fw | CGAGCATAGAATTTCTCTCCTTATC | SS2 CO rv2 (in *SS2*-*HA*) | GGTTTGTAAGGTGGGTCAACAG | - | 1928 | 1928 |  |  |
|  | X-4 down out rv | CGAGCATAGAATTTCTCTCCTTATC | X-4 down out rv | GGACGGTACGTTGACCAGAGATTG | 1336 | 6220 | 4827 |  |  |
|  | DR fw | TGTACAGACGCGTGTACGCATG | X-4 down out rv | GGACGGTACGTTGACCAGAGATTG | - | 2410 | 1017 |  |  |
| **pXI-2_ P*_GAL1_*-*SS2*-*HA*** | XI-2 up out fw | CGTCCAAATAATTCCCTTCGACAGG | SS2 CO rv2 | GGTTTGTAAGGTGGGTCAACAG | - | 1862 | 1862 |  |  |
|  | XI-2 up out fw | CGTCCAAATAATTCCCTTCGACAGG | XI-2 down out rv | ATTTTGGCATTTCCCTGCAGCTGATC | 1383 | 6210 | 4817 |  |  |
|  | DR fw | TGTACAGACGCGTGTACGCATG | XI-2 down out rv | ATTTTGGCATTTCCCTGCAGCTGATC | - | 2466 | 1073 |  |  |
| **pGSY2_ P*_GAL1_*-*SS3*** | GSY2 up out fw | GTGTGTTGAGCAAAGCACGCCATCAC | ss3 seq rv1 (in *SS3*) | CGGTTGAAGCAATTCACGTCTC | - | 2243 | 2243 |  |  |
|  | GSY2 up out fw | GTGTGTTGAGCAAAGCACGCCATCAC | GSY2 down out rv | GAGACCCATTCTTTTCCAGTCCAG | 2467 | 7042 | 5649 |  |  |
|  | DR fw | TGTACAGACGCGTGTACGCATG | GSY2 down out rv | GAGACCCATTCTTTTCCAGTCCAG | - | 2355 | 962 |  |  |
| **pGSY2_ P*_GAL1_*-*SS4*** | GSY2 up out fw | GTGTGTTGAGCAAAGCACGCCATCAC | SS4 seq rv1 (in *SS4*) | AACCACGTTGTTGTCGCATC | - | 1485 | 1485 |  |  |
|  | GSY2 up out fw | GTGTGTTGAGCAAAGCACGCCATCAC | GSY2 down out rv | GAGACCCATTCTTTTCCAGTCCAG | 2467 | 7066 | 5673 |  |  |
|  | DR fw | TGTACAGACGCGTGTACGCATG | GSY2 down out rv | GAGACCCATTCTTTTCCAGTCCAG | - | 2355 | 962 |  |  |
|  | 805 (in SS4) | CAGATGATCGCTATGAGATATGG | GSY2 down out rv | GAGACCCATTCTTTTCCAGTCCAG | - | 2930 | 1537 |  |  |
| **pXII-1_ P*_GAL1_*-*SS4*** | XII-1 up out fw | GATTGGCTTGATGATACCTTCC | SS4 seq rv1 (in *SS4*) | AACCACGTTGTTGTCGCATC | - | 1447 | 1447 |  |  |
|  | XII-1 up out fw | GATTGGCTTGATGATACCTTCC | XII-1 down out rv | CGCTCATCTAGTGAGAATGTCAC | 1488 | 7065 | 5672 |  |  |
|  | DR fw | TGTACAGACGCGTGTACGCATG | XII-1 down out rv | CGCTCATCTAGTGAGAATGTCAC | - | 2370 | 977 |  |  |
|  | 805 (in SS4) | CAGATGATCGCTATGAGATATGG | XII-1 down out rv | CGCTCATCTAGTGAGAATGTCAC | - | 2967 | 1574 |  |  |
| **pXII-5_ P*_GAL1_*-*BE1*** | XII-5 up out fw | GAGGACGAAGAAGGCCTGCAATTC | BE1 seq rv (in *BE1*) | GGTTCTTCACCCTCTCTTAACT | - | 1700 | 1700 |  |  |
|  | yeast fw2 | GTACCCAATTCGCCCTATAG | XII-5 down out rv | AAGGTTGTTGATCAGTGTTCATGG |  | 921 | 921 |  |  |
| **pGLC3_ P*_GAL1_*-*BE1*** | GLC3 up out fw | CATACTATACCACACGTACGAC | BE1 seq rv (in *BE1*) | GGTTCTTCACCCTCTCTTAACT | - | 1664 | 1664 |  |  |
|  | yeast fw2 | GTACCCAATTCGCCCTATAG | GLC3 down out rv | AGTACCAGCTTTTTCGACACC | - | 752 | 752 |  |  |
| **pXII-2_ P*_GAL1_*-*BE2*** | XII-2 up out fw | GAGGACGAAGAAGGCCTGCAATTC | BE2 rv new | AAACCTCTGGGATCATCATCAAG | - | 1465 | 1465 |  |  |
|  | XII-2 up out fw | GAGGACGAAGAAGGCCTGCAATTC | XII-2 down out rv | AAGGTTGTTGATCAGTGTTCATGG | 1296 | 6196 | 4803 |  |  |
|  | 966 (in BE2) | GGCAATGCAACATCTTGAAGAGAA | XII-2 down out rv | AAGGTTGTTGATCAGTGTTCATGG | - | 2901 | 1508 |  |  |
| **pGLC3_ P*_GAL1_*-*BE2*** | GLC3 up out fw | CATACTATACCACACGTACGAC | BE2 rv new (in *BE2*) | AAACCTCTGGGATCATCATCAAG | - | 1365 | 1365 |  |  |
|  | GLC3 up out fw | CATACTATACCACACGTACGAC | GLC3 down out rv | AGTACCAGCTTTTTCGACACC | 2455 | 6092 | 4699 |  |  |
|  | 966 BE2 fw | GGCAATGCAACATCTTGAAGAGAA | GLC3 down out rv | AGTACCAGCTTTTTCGACACC | - | 2897 | 1504 |  |  |
| **pXI-2_ P*_GAL1_*-*BE2*** | XI-2 up out fw | CGTCCAAATAATTCCCTTCGACAGG | BE2 rv new (in *BE2*) | AAACCTCTGGGATCATCATCAAG | - | 1476 | 1476 |  |  |
|  | XI-2 up out fw | CGTCCAAATAATTCCCTTCGACAGG | XI-2 down out rv | ATTTTGGCATTTCCCTGCAGCTGATC | 1383 | 6256 | 4863 |  |  |
|  | 966 BE2 fw | GGCAATGCAACATCTTGAAGAGAA | XI-2 down out rv | ATTTTGGCATTTCCCTGCAGCTGATC | - | 2950 | 1557 |  |  |
| **pGLC3_ P*_GAL1_*-*BE3*** | GLC3 up out fw | CATACTATACCACACGTACGAC | BE3 rev (in *BE3*) | CCTTCCCAGAAGAACGAGAGTCC | - | 1307 | 1307 |  |  |
|  | GLC3 up out fw | CATACTATACCACACGTACGAC | GLC3 down out rv | AGTACCAGCTTTTTCGACACC | 2455 | 6281 | 4888 |  |  |
|  | DR fw | TGTACAGACGCGTGTACGCATG | GLC3 down out rv | AGTACCAGCTTTTTCGACACC | - | 2413 | 1020 |  |  |
| **pXI-2_ *ISA1*-P*_GAL10_*-P*_GAL1_*-*ISA2*** | XI-2 up out fw | CGTCCAAATAATTCCCTTCGACAGG | ISA1 seq rv 1 (in *ISA1*) | ATGGGATGCGGGTGGGC | - | 1866 | 1866 |  |  |
|  | XI-2 up out fw | CGTCCAAATAATTCCCTTCGACAGG | XI-2 down out rv | ATTTTGGCATTTCCCTGCAGCTGATC | 1383 | 9019 | 7626 |  |  |
|  | DR fw | TGTACAGACGCGTGTACGCATG | XI-2 down out rv | ATTTTGGCATTTCCCTGCAGCTGATC | - | 2466 | 1073 |  |  |
|  | ISA2 seq fw2 (in ISA2) | CTC CGA ACA TAC CTC TGG | XI-2 down out rv | ATTTTGGCATTTCCCTGCAGCTGATC | - | 4181 | 2788 |  |  |
| **P*_GAL1-_glgC-TM-HA*** | pGAL1 seq fw (in P*_GAL1_)* | GGGGTAATTAATCAGCGAAGCG | GlgCTM rv (in g*lgC*-*TM*) | AATGACCAGCCGCGCTGAATGTGC | - | 477 | 477 |  |  |
| **P*_GAL1_*-*SS1*** | pGAL1 seq fw (in P*_GAL1_)* | GGGGTAATTAATCAGCGAAGCG | SS1 seq rv (in *SS1*) | CCTTCAACAGTAGTGATTTCCC | - | 1236 | 1236 |  |  |
| **P*_GAL1_*-*SS2*-*HA*** | pGAL1 seq fw (in P*_GAL1_)* | GGGGTAATTAATCAGCGAAGCG | SS2 CO rv2 (in *SS2*) | GGTTTGTAAGGTGGGTCAACAG | - | 689 | 689 |  |  |
| **P*_GAL1_*-*SS3*** | pGAL1 seq fw (in P*_GAL1_)* | GGGGTAATTAATCAGCGAAGCG | SS3 seq rv1 (in *SS3*) | CGGTTGAAGCAATTCACGTCTC | - | 1012 | 1012 |  |  |
| **P*_GAL1_*-*SS4*-*FLAG*** | pGAL1 seq fw (in P*_GAL1_)* | GGGGTAATTAATCAGCGAAGCG | SS4 seq rv (in *SS4*) | AACCACGTTGTTGTCGCATC | - | 254 | 254 |  |  |
| **P*_GAL1_*-*BE2*** | pGAL1 seq fw (in P*_GAL1_)* | GGGGTAATTAATCAGCGAAGCG | BE2 rv new (in *BE2*) | AAACCTCTGGGATCATCATCAAG | - | 303 | 303 |  |  |
| **P*_GAL1_*-*BE3*** | pGAL1 seq fw (in P*_GAL1_)* | GGGGTAATTAATCAGCGAAGCG | BE3 rev (in *BE3*) | CCTTCCCAGAAGAACGAGAGTCC | - | 245 | 245 |  |  |
| ***ISA1*- P*_GAL10_*- P*_GAL1_*-*ISA2*** | pGAL1 seq fw (in P*_GAL1_)* | GGGGTAATTAATCAGCGAAGCG | ISA2 seq rv (in *ISA2*) | CACCACTAAGTTCCAAAGATGAGAC | - | 524 | 524 |  |  |

**Supplementary File 1E.** Mutant plant alleles and primers used for their identification. The fwd (forward) and rev (reverse) primers were used for the amplification of wild-type alleles. To amplify the mutated allele, the primers depicted in bold were used. WS, Wassilewskija;

| **Gene/AGI code** | **Mutation type, position** | **Mutant allele, Line identifier (other name)** | **Primers used to select mutant alleles (shown 5’to 3’)** | **Eco-type** | **Reference** |
| --- | --- | --- | --- | --- | --- |
| *SS1*  At5g24300 | T-DNA  Insertion in intron 1 | *ss1-*1,  Genoplante_203C08 | **Fwd**: TTTCCGTCCGATCGCCAGTCTC  Rev: TACGCCAAAGTCAGCCATTACAA  **T-DNA**: CTACAAATTGCCTTTTCTTATCGAC | WS | Delvallé et al., 2005 |
| *SS2* At3g01180 | T-DNA  Insertion in exon 8 | *ss2-*3,  Genoplante_549A11 | Fwd: CCTAGTGGTGGAAAATTAGGGG  **Rev**: AACCGAGAATCCAACCCATC  **T-DNA**: CTACAAATTGCCTTTTCTTATCGAC | WS | Zhang et al., 2008 |
| *SS3*  At1g11720 | T-DNA  Insertion in exon 12 | *ss3-*3, Genoplante_117H05 | **Fwd**: TTACGCGCTTAACACACCAGAAG  Rev: ATTCATCTTAGAGCTTCCATTTTA  **T-DNA**: CTACAAATTGCCTTTTCTTATCGAC | WS | Szydlowski et al., 2009 |
| *SS4*  At4g18240 | T-DNA  Insertion in intron 2 | *ss4-2*  Genoplante_559H08 | Fwd: AACCCATGGATTAGCAGGAA **Rev:** CAAATGGGAAATGAAAGGAAC **T-DNA:** CTGATACCAGACGTTGCCCGCATAA | WS | Roldán et al., 2007 |

**Supplementary References**

**Mikkelsen, M.D., Buron, L.D., Salomonsen, B., Olsen, C.E., Hansen, B.G., Mortensen, U.H., and Halkier, B.A.** (2012). Microbial production of indolylglucosinolate through engineering of a multi-gene pathway in a versatile yeast expression platform. Metab. Eng. **14**: 104–111.
